# Full-Face Allograft Retrieval in a Multiple-Organ Donation in a Maastricht III Type Donor

**DOI:** 10.3390/jcm14051682

**Published:** 2025-03-02

**Authors:** Juan P. Barret, Cristina Dopazo, Alberto Sandiumenge, Itxarone Bilbao, Ramón Charco

**Affiliations:** 1 Department of Plastic Surgery and Burns, Vall d’Hebron Barcelona Hospital Campus Paseo Vall d’Hebron 119-129, 08035 Barcelona, Spain; 2 Department of Surgery, School of Medicine, Universitat Autònoma de Barcelona, 08193 Bellaterra, Spain; cristina.dopazo@vallhebron.cat (C.D.); alberto.sandiumenge@vallhebron.cat (A.S.); itxarone.bilbao@vallhebron.cat (I.B.); rcharco@gmail.com (R.C.); 3 Department of Hepatic and Biliary Surgery and Transplants, Vall d’Hebron Barcelona Hospital Campus, 08035 Barcelona, Spain; 4 Department of Transplant Coordination, Vall d’Hebron Barcelona Hospital Campus, 08035 Barcelona, Spain

**Keywords:** VCA, face transplantation, donation, DCD, asystole, Maastricht Type Donor

## Abstract

**Background:** Donation after circulatory death (DCD) has emerged as a potential source of transplantable organs. To date, there have been no reports of face procurement in AD, and “face first” with ex situ perfusion has become the gold standard technique for obtaining facial allografts in most centres. **Objectives:** We report a case of successful total face and kidney transplantation from a 47-year-old male AD donor. **Methods:** Immediately after confirmation of death, the “rapid recovery” technique was performed and a cannula was placed in the ascending aorta for in situ perfusion of the facial allograft simultaneously with the abdominal team. **Results:** The total ischaemic time from donor cardiac death to face reperfusion in the recipient was 5.5 h. Excellent renal and facial allograft function was reported.

## 1. Introduction

The first successful partial face transplant was performed in France in November 2005 [1]. As of October 2024, a total of 53 face transplants have been performed worldwide [2,3], including the first full face transplant in 2010 [4]. Face Vascularised Composite Allotransplantation (FVCA) is one of the most recent additions to the reconstructive ladder in plastic and reconstructive surgery, making FVCA a true paradigm shift in craniofacial reconstruction and facial plastic surgery [5]. It allows the resection of tumours and deformed facial tissues and craniofacial surgery that would not otherwise be possible; it uses healthy facial tissue transplanted from donors to reconstruct the damaged or absent facial tissues with original tissues, making it possible to achieve the best possible functional and aesthetic results in these challenging injuries.

From an organisational and logistical point of view, there are significant differences in the donation process when face retrieval is included. Different approaches have been used when considering face retrieval in a multiorgan donation: procurement of the face with intact circulation either at the beginning of the procurement [1,6], synchronous in situ dissection together with abdominal and thoracic organs [7] or procurement of the face allograft without intact circulation at the end of the procurement [3]. In the past, most facial transplant teams have advocated for retrieval from a heart-beating donor with intact circulation, either at the beginning of the operation or during a synchronous procurement.

Herein we present our experience with the retrieval of facial allograft in a controlled asystole donor (Maastritch III). The peculiarity of the case that we describe here is that the donor was a controlled donation after circulatory death (cDCD). The “rapid recovery” (RR) technique was performed for kidneys and complex facial Vascularised Composite Allotransplantation (VCA) procurement and it seems that the effect of warm ischemia during the hypotensive phase after the withdrawal of life-sustaining therapy (WLST), which characterises AD, did not interfere in the further good results.

## 2. Case Report

The operation was performed in February 2015. A 47-year-old man, an active smoker with a history past history of harmful alcohol use, dislipemic and dilated cardiomyopathy with severe systolic dysfunction and severe mitral regurgitation with an implantable cardioverter defibrillator, was admitted to the Cardiac Intensive Care Unit after sudden cardiac arrest which was reversed after 30 min of cardiopulmonary resuscitation. Seventy-two hours later, with no expectation of meaningful survival as determined by the patient’s care team due to devastating anoxic encephalopathy, withdrawal of ventilatory and organ perfusion support was agreed upon between the care team and the patient’s family. Following the patient’s wishes, as determined by discussion with his family, a DCD procedure was considered. Neither the lungs nor the liver were considered optimal for donation based on past medical history and radiological findings. However, it was considered to proceed with facial VCA plus procurement of kidneys from the currently controlled AD.

Before WLST, the patient was sterilised and prepped in the usual way. WLST took place in the operating theatre and death was certified according to Spanish law [8] (5 min of non-touch period) after 18 min of functional warm ischaemia time (22 min total warm ischaemia). After complete heparinisation of the donor, a “rapid recovery” technique was performed and rapid thoracotomy and laparotomy were performed. Avoidance of cerebral reperfusion after pronouncement of death declaration is mandatory. In this case scenario, a “rapid recovery” technique was used for organ procurement, avoiding the reperfusion of the brain with oxygenated blood.

The abdominal aorta was cannulated with a 22F cannula and the descending thoracic aorta was clamped. The abdominal organs were perfused with 3 litres of cold UW solution (Bel-Gen^R^ solution, Institut George Lopez, Lissieu, France) and the right atrium was opened for drainage. Simultaneously, in the thoracic field, the origin of the ascending aorta was encircled and clamped while a 14F cannula was placed anterior to the origin of the brachiocephalic trunk. The aorta was further cross-clamped in the descending part beyond the origin of the left subclavian artery. Immediately, the facial VCA was perfused with 2 L of cold UW solution (Bel-Gen^R^ solution, Institut George Lopez, Lissieu, France) via the carotid-jugular system, followed by a superior vena cava transection to allow drainage of the facial venous system.

The kidneys were procured in a standard manner while the cannula in the ascending aorta was secured with a purse-string suture and the cold perfusion of the facial VCA was maintained slowly throughout the 4 h of dissection to avoid rewarming.

Dissection and procurement of the kidneys and the facial allograft were performed synchronously.

**Figure 1:**
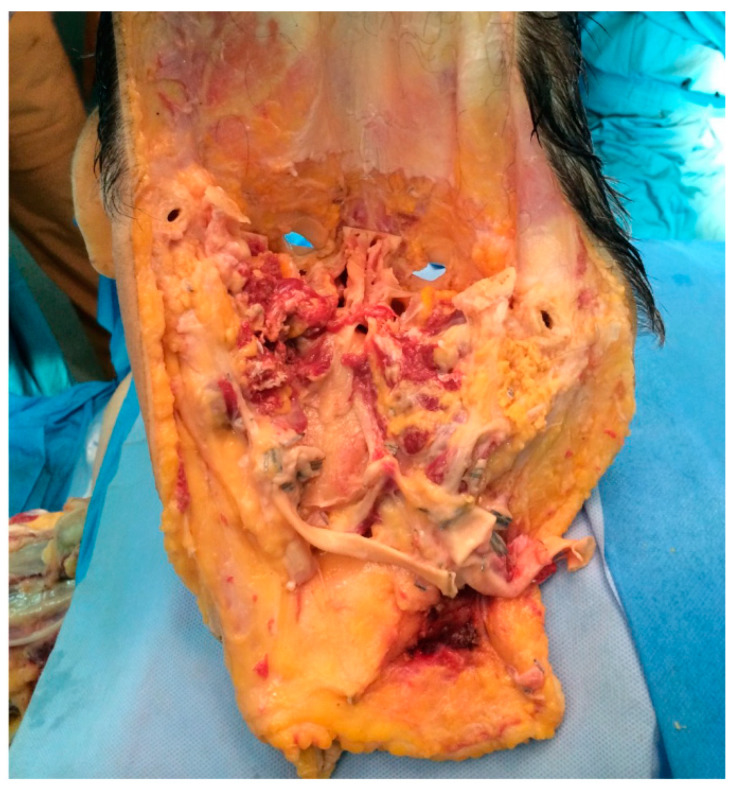
Posterior view of the full-face allograft at the end of the retrieval under a controlled asystole donation. Note that all vessels, nerves and muscles have been identified. The allograft consisted of a full face procurement including the mandible, part of the temporal bone, maxillary and zigomas.

A complete facial allograft was obtained including the mandible, temporomandibular joints, maxilla and zygomatic bones. The area of skin, soft tissue and muscle included in the allograft extended from the vertex to the mid-neck and the retro-auricular area bilaterally. Dissection started from the vertex to the orbital area and laterally to the zygoma. All sensory nerves (supraorbital, infraorbital and dental nerve) and all five branches of the facial nerve were identified and divided at their origin. Osteotomies were made in the maxilla, in the zygoma and in the glabella using a Lefort III osteotomy. Dissection proceeded inferiorly to the neck to identify and dissect the external and internal jugular veins and the external carotid artery to the common carotid artery. The internal carotid artery and the internal jugular vein were ligated. At this point, the facial allograft was pedicled to the cervical vessels, which were cut to completely free the allograft (Figure 1). Dissection was performed with needle point electrocautery and major vessels were ligated for haemostasis. The face was then packed in a sterile container in the usual way and sent to the recipient’s operating theatre. The entire procedure took 4 h. At the end of the procedure, the donor was fitted with a custom-made face mask [9,10].

The transplantation procedure in the recipient followed the step-by-step approach as the previously described techniques for facial transplantation [4]. The indication for facial allotransplantation was a massive arteriovenous malformation of the central area of the face and neck. The patient had suffered massive life-threatening haemorrhages in the past and all medical and surgical treatments were unsuccessful. Complete resection of the malformation was considered and the only reconstructive alternative to such a massive resection was a vascularised composite allograft of the face and neck.

The first step in the recipient was to revascularise the facial allograft to ensure correct vascularisation of the graft prior to the resection of the recipient’s facial tissue. To this end, an end-to-end anastomosis of both external carotid arteries was performed. Venous return was achieved with an end-to-side anastomosis between the internal jugular veins bilaterally. Induction immunosuppression consisted of rabbit antithymocyte globulin rATG (Thymoglobulin; Sanofi, Paris, France) with a target total dose of 9 mg/kg body weight plus extended-release tacrolimus once daily with a target drug trough level of 10–12 ng/mL, mycophenolate mofetil at two 1 g per day and prednisolone tapered according to our police. The total cold ischaemia time of the facial allograft was 5.5 h.

**Figure 2:**
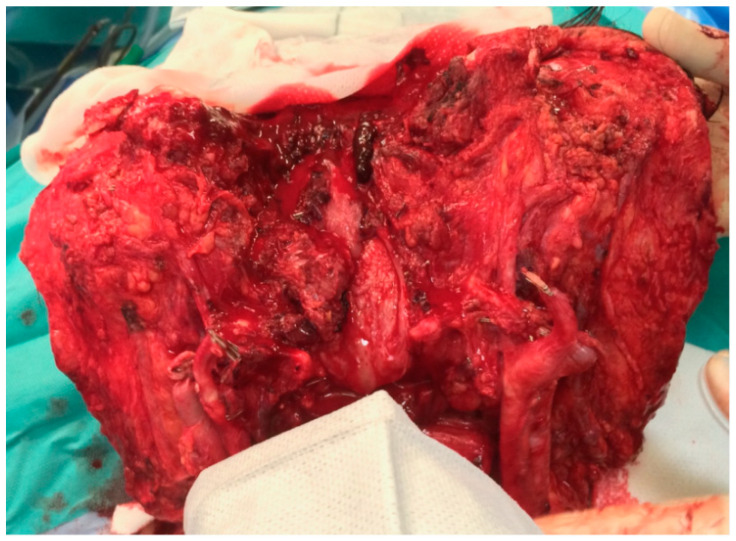
Complete vascularization was achieved in the recipient. There was not any ischemic area with excellent outcome.

The entire facial allograft showed excellent and complete arterial perfusion with correct venous return. There were no ischaemic areas of skin, soft tissue or bone. The bleeding was similar to that observed in facial allografts obtained from heart-beating multiorgan donations with intact circulation. (Figure 2) Haemostasis was achieved with electrocautery, bipolar coagulation and ligation of any open vessels. The remainder of the transplantation was uneventful. The patient made a full recovery after the operation without any wound problems in the facial allograft. The long-term function of the facial allograft was excellent. All facial muscles have regained normal function. Normal sensation in the trigeminal nerves preceded the normal function of the facial muscles. To date (10-year follow-up), no evidence of chronic rejection or other functional or throphic abnormalities has been observed. Current immunosuppressive therapy includes prednisone and tacrolimus. The current outcome is similar to that of previous facial allografts procured with intact circulation.

Kidney function was also reported to be excellent.

## 3. Discussion

Full-face allograft procurement in a controlled asystole donation (Maastrict III), including abdominal organs proved to be a feasible and safe approach. In our hands, the logistics and strategy of multiple-organ donation, including internal organs, was easier than facial allograft procurement in a multiple-organ donation in a heart-beating donor. The operation (procurement) was shorter than in other previous reports (4.5 h versus a total of 7.5 h) [7] without the need for blood products during the procurement procedure. Some VCA transplant centres advocate limiting the ischaemia time to less than 4 h in order to protect the muscles contained in the facial allografts [11,12]. However, there are reports of successful facial transplantation with the retrieval of facial allografts at the end of multiorgan procurement with longer cold ischaemia times [13]. This was also our experience. The allograft showed complete viability with excellent facial function after nerve recovery. To ensure a safe reconstructive procedure after facial resection, we recommend a conservative approach similar to that proposed by Meningaud [14]. Revascularisation of the facial allograft at the beginning of the recipient operation allows for shorter ischaemia time and the viability of the graft can be assessed before the recipient’s face is resected. Indocyanine green fluorescence angiography [15] may also be used during the procurement phase and after the revascularisation of the graft. It allows the tissue to be examined under polarised light. All areas of good vascularisation are stained by the drug, whereas areas of poor or no perfusion appear dark and should be discarded. We recommend that this technique be used for all future facial transplants.

The perioperative anaesthetic challenges of multiorgan procurement including a full-face allograft are significant in both the donor and the recipient [16]. DCD face procurement and face procurement at the end of multiorgan donation in a brain-dead donor reduce the risk of complications during multiorgan donation and speed up the procedure. The need for blood products is minimal and the risk of instability and injury to otherwise viable vital organs is negligible. There is also no need to perform a tracheostomy to obtain a facial allograft.

A successful face transplantation programme requires a sound research protocol, a solid infrastructure, expert staff and adequate funding [17,18]. Organ procurement including face retrieval is challenging. Organ shortage is a global problem and remains a major concern. It is multifactorial and requires a comprehensive approach [19]. DCD of organs and faces has been shown to be safe and effective with excellent outcomes. This approach aims to increase donation opportunities and the pool of available organs and vascularised composite allografts.

Novel imaging techniques, 3D model printing, 3D simulation and intraoperative navigation are also new tools in FVCA and in facial plastic surgery. The use of such technologies improves predictability and safety by better matching the facial bones. Manninen et al. [20] reported the feasibility of 3D planning in two facial transplants with almost exact bone reconstruction in facial transplantation. Holographic virtual planning is also a very promising technology for use in complex craniofacial surgery [21]. It has been shown to be time efficient with unlimited preoperative surgical rehearsal, and potential for intraoperative surgical guidance. Other promising technologies to be explored to improve digital planning and execution of FVCA include artificial intelligence tools such as deep learning and mathematical models with pixel noise reduction to allow for more accurate 3D and image reconstruction and surgical preparation in both donor and recipient [22,23].

In conclusion, we believe that face procurement in a DCD is a valuable addition to the options for face allograft procurement in a multiorgan donation. It will also increase the pool of donors available for vascularised composite tissue allografts.

## Data Availability

All data regarding this case report is available in the Electronic Hospital Clinical History of the Patient. According to the European Data Protection Law any confidential information or personal data cannot be disclosed or released to third parties. No personal data are included in this report since it is not relevant for the scientific purpose of the manuscript.

## Abbreviation

AD	Asystole Donation
DCD	Donation after Cardiac Death
RR	Rapid Recovery
rATG	rabbit Antithymocyte Globulin
UW	University of Wisconsin
VCA	Vascularized Composite Allotransplantation
WLST	Withdrawal of Life Sustaining Therapy
FVCA	face Vascularized Composite Allotransplantation
